# A166 SIMPLENESS: A QUALITATIVE DESCRIPTION STUDY EXPLORING PATIENT PERSPECTIVES ON THE BARRIERS AND FACILITATORS OF USING DIGITAL HEALTH TOOLS TO SELF-MANAGE INFLAMMATORY BOWEL DISEASE

**DOI:** 10.1093/jcag/gwae059.166

**Published:** 2025-02-10

**Authors:** L J Ajibulu, K Chappell, C Seow, K J Goodman, K Wong

**Affiliations:** Medicine, University of Alberta Faculty of Medicine & Dentistry, Edmonton, AB, Canada; Medicine, University of Alberta Faculty of Medicine & Dentistry, Edmonton, AB, Canada; University of Calgary Cumming School of Medicine, Calgary, AB, Canada; Medicine, University of Alberta Faculty of Medicine & Dentistry, Edmonton, AB, Canada; Medicine, University of Alberta Faculty of Medicine & Dentistry, Edmonton, AB, Canada

## Abstract

**Background:**

Inflammatory Bowel Disease (IBD) is a chronic condition that requires lifelong management and frequent interaction with healthcare providers. Digital health tools have the potential to enhance disease management by offering real-time data and improving care coordination. However, there is limited evidence on patient perspectives regarding the barriers and facilitators to using these tools effectively.

**Aims:**

This study aimed to explore patient perspectives on the use of digital health tools for managing IBD, identifying key barriers and facilitators that influence their adoption and sustained use.

**Methods:**

Using a qualitative description methodology, participants with a confirmed diagnosis of IBD were purposively recruited from multiple clinics in Alberta, Canada. Data was collected through virtual semi-structured interviews until thematic saturation was reached. Participants were introduced to “*MyIBDToolkit*,” a digital health tool in development, designed for seamless communication and data sharing between patients and providers, integrated into an electronic health record (EHR) patient portal to support self-management. Thematic analysis was used to identify key themes within the interviews.

**Results:**

Thematic saturation was achieved with 18 interviews. Participants expressed varied views on self-management and digital disease management. Most were receptive to using digital health tools, highlighting benefits like enhanced monitoring and streamlined care. However, they noted concerns about sustainability, data entry burden, and privacy as significant barriers. Some participants were also skeptical about healthcare provider use of these tools.

**Conclusions:**

While participants generally viewed digital health tools as beneficial for self-managing IBD, usability, engagement, and privacy challenges must be addressed for successful implementation. These findings underscore the importance of incorporating patient perspectives in developing and integrating digital health interventions for chronic disease management.

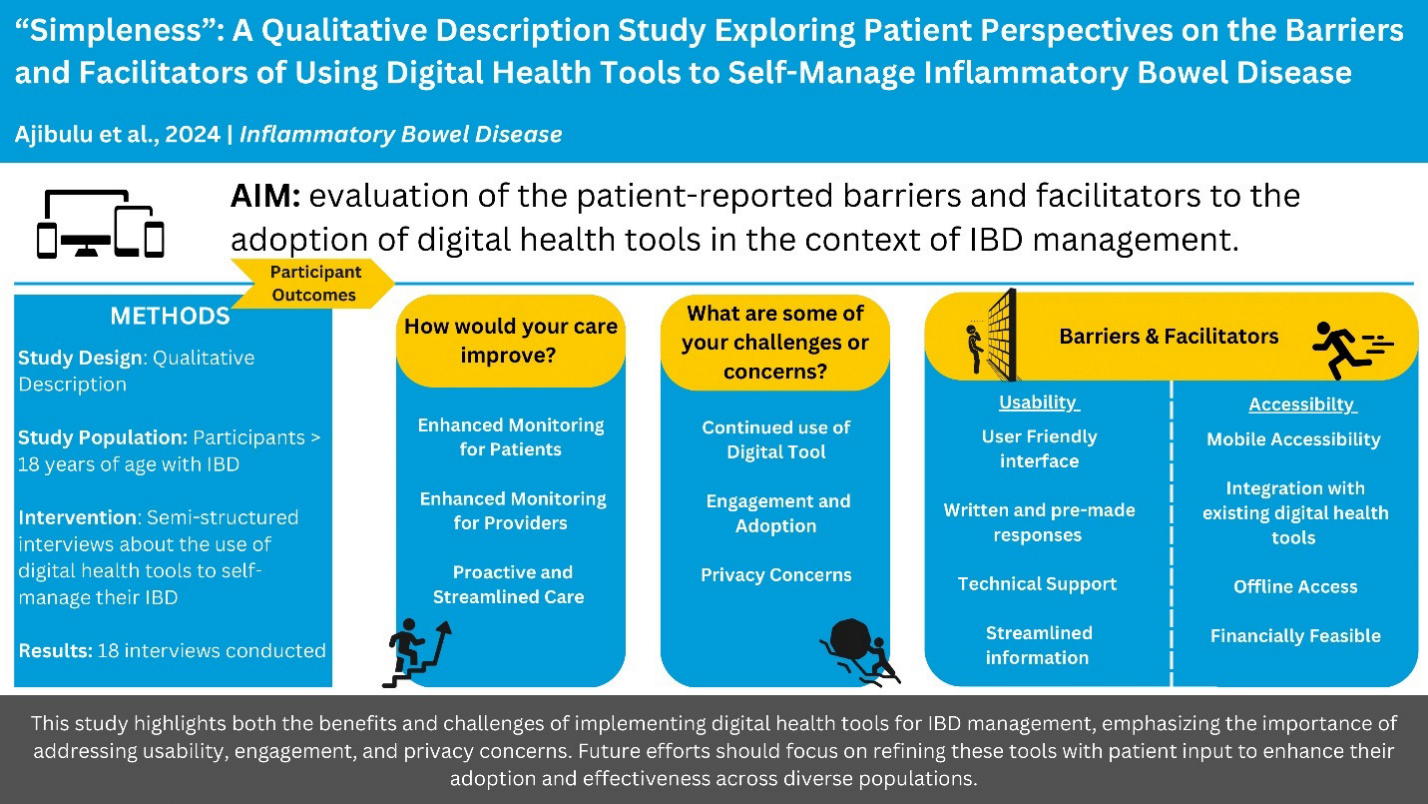

This graphical abstract highlights the key findings from semi-structured interviews with IBD patients, focusing on the potential benefits and challenges of digital health tools for disease management. The study identified improved care monitoring, streamlined care, and usability features as facilitators, while concerns about privacy, continued use, and adoption hindered engagement.

**Funding Agencies:**

Alberta Innovates Partnership for Research and Innovation in the Health System 7 (PRIHS 7)

